# Molecular Epidemiology of *Clostridioides difficile* Colonization in Families With Infants

**DOI:** 10.1093/ofid/ofae299

**Published:** 2024-06-10

**Authors:** Christine Marlow, Jason A Clayton, Nori Minich, Gregory Golonka, Lynn Maruskin, Annette L Jencson, Jennifer M Hailes, Hosoon Choi, Piyali Chatterjee, Munok Hwang, Chetan Jinadatha, Jennifer L Cadnum, Curtis J Donskey, Philip Toltzis

**Affiliations:** Department of Pediatrics, Rainbow Babies and Children's Hospital, Cleveland, Ohio, USA; Department of Pediatrics, Rainbow Babies and Children's Hospital, Cleveland, Ohio, USA; Department of Pediatrics, Rainbow Babies and Children's Hospital, Cleveland, Ohio, USA; Kids in the Sun, Strongsville, Ohio, USA; Kids in the Sun, Strongsville, Ohio, USA; Infectious Diseases Section (CJD) and Research Service (ALJ, JMH, JLC), Louis Stokes Cleveland Veterans Affairs Medical Center, Cleveland, Ohio, USA; Infectious Diseases Section (CJD) and Research Service (ALJ, JMH, JLC), Louis Stokes Cleveland Veterans Affairs Medical Center, Cleveland, Ohio, USA; Department of Medicine (CJ) and Department of Research (HC, PC, MH), Central Texas Veterans Healthcare System, Temple, Texas, USA; Department of Medicine (CJ) and Department of Research (HC, PC, MH), Central Texas Veterans Healthcare System, Temple, Texas, USA; Department of Medicine (CJ) and Department of Research (HC, PC, MH), Central Texas Veterans Healthcare System, Temple, Texas, USA; Department of Medicine (CJ) and Department of Research (HC, PC, MH), Central Texas Veterans Healthcare System, Temple, Texas, USA; Infectious Diseases Section (CJD) and Research Service (ALJ, JMH, JLC), Louis Stokes Cleveland Veterans Affairs Medical Center, Cleveland, Ohio, USA; Infectious Diseases Section (CJD) and Research Service (ALJ, JMH, JLC), Louis Stokes Cleveland Veterans Affairs Medical Center, Cleveland, Ohio, USA; Department of Pediatrics, Rainbow Babies and Children's Hospital, Cleveland, Ohio, USA

**Keywords:** *Clostridioides difficile*, epidemiology, gastrointestinal colonization

## Abstract

**Background:**

Community-associated *Clostridioides difficile* infection is a major public health hazard to adults and older children. Infants frequently excrete toxigenic *C difficile* asymptomatically in their stool, but their importance as a community reservoir of *C difficile* is uncertain.

**Methods:**

Families of healthy infants were recruited at the baby's 4-month well child visit and were followed longitudinally until the baby was approximately 9 months old. Babies and mothers submitted stool or rectal swabs every 2 weeks that were cultivated for *C difficile*; fathers’ participation was encouraged but not required. *Clostridioides difficile* isolates were strain-typed by fluorescent polymerase chain reaction ribotyping and by core genome multilocus sequence typing, and the number of families in whom the same strain was cultivated from >1 family member (“strain sharing”) was assessed.

**Results:**

Thirty families were enrolled, including 33 infants (3 sets of twins) and 30 mothers; 19 fathers also participated. *Clostridioides difficile* was identified in 28 of these 30 families over the course of the study, and strain sharing was identified in 17 of these 28. In 3 families, 2 separate strains were shared. The infant was involved in 17 of 20 instances of strain sharing, and in 13 of these, the baby was identified first, with or without a concomitantly excreting adult. Excretion of shared strains usually was persistent.

**Conclusions:**

*Clostridioides difficile* strain sharing was frequent in healthy families caring for an infant, increasing the likelihood that asymptomatically excreting babies and their families represent a reservoir of the organism in the community.

## Teaser

The role of asymptomatically excreting infants in spreading *Clostridioides difficile* in the community is uncertain. We longitudinally followed 30 families caring for healthy infants and found that *C difficile* strains frequently were shared among babies and their parents.

*Clostridioides difficile*, once a pathogen encountered primarily in inpatient facilities, has emerged as a prominent cause of diarrhea outside the hospital. Recent surveys indicate that community-associated *C difficile* infection (CA-CDI) accounts for nearly half of all *C difficile* enteritis in the United States (US) [1]. The identification of reservoirs of infection in the hospital, namely, persistently contaminated inanimate surfaces and caregiver hands [2], has resulted in the development of effective measures to lessen transmission. By contrast, the reservoirs of the organisms implicated in community-acquired infection are uncertain, but their identification is critical in developing rational strategies to prevent their spread.

Infants asymptomatically excrete *C difficile* throughout the first 1–2 years of life [3], with colonization exceeding 50% at 6 months of age [4], but the role of colonized infants in sustaining the presence of *C difficile* in the community has not been clearly demonstrated. The putative epidemiologic importance of asymptomatically excreting infants can be reasoned in both directions. On one hand, the daily care of babies requires close contact by their adult caregivers, and activities such as diaper changes and bathing present abundant opportunities for transmission of *C difficile* to other household members. On the other hand, the integrity of the gastrointestinal microbiome of most parents may represent a barrier to spread beyond the baby, diminishing or eliminating the epidemiological role of infants in CA-CDI. The aim of the current study, therefore, was to measure the frequency in which *C difficile* excreted by infants can be identified in a parent, as a step in determining the importance of infants in the spread of *C difficile* in the community.

## MATERIALS AND METHODS

### Subject Recruitment

Families of healthy infants were recruited at their child's 4-month well child visit at a pediatric office located in a demographically diverse suburb of Cleveland, Ohio. Infants’ and mothers’ participation were required; fathers were encouraged to participate but their inclusion was not mandatory. Families were instructed to mail a soiled infant diaper via the US Postal Service to a research microbiology laboratory every 2 weeks until the child was approximately 8–9 months of age, resulting in a total of 8 study time points over 4 months. Simultaneous adult rectal specimens were collected using commercial diaper wipes that were sent in the same mailer as the diapers but in separate sealed plastic bags.

### Culture and Strain Typing

Samples were serially diluted and inoculated onto *C difficile* selective media under anaerobic conditions, as previously described [5]. Additionally, samples were broth-enriched overnight in media selective for *C difficile* (limit of detection 1 log_10_ organisms) [6] before plating. Organisms cultivated by either method were identified by colony morphology, and toxin production was assessed using a commercial enzyme immunoassay applied to the isolate (Alere, Abbott Rapid Diagnostics). When colony morphology was equivocal, the isolate was assayed for glutamine dehydrogenase production (Quick Check Complete, Techlab). All organisms then were stored at −80°C until further testing.

Isolates were strain-typed employing 2 methods, namely, fluorescent polymerase chain reaction (PCR) ribotyping and whole genome sequence (WGS)–based core genome multilocus sequence typing (cgMLST). Fluorescent PCR ribotyping was achieved using previously described methods [7]. In short, bacterial genomic DNA was extracted and polymorphic segments included in the rRNA operon were amplified. Amplicons then were size-separated by capillary electrophoresis and fluoresced to produce distinct chromatograms. PCR ribotype was assigned by comparing the pattern of the resulting peaks to an established database.

For WGS-based cgMLST [8] (see Supplementary Table 1 for detailed WGS methodology), stored organisms were cultivated on anaerobic blood agar plates overnight. After DNA extraction, a DNA library was prepared using the Nextera DNA Prep Kit (Illumina, San Diego, California), and paired-end reads (2 × 150 bp) were generated using the Illumina NextSeq mid output reagent kit and NextSeq 550 instrument (Illumina, San Diego, California). De novo assembly was completed using SPAdes genome assembler on a cloud-based platform (Applied Maths, Austin, Texas). After quality assurance to ensure the accuracy of the calls, the isolates were assigned multilocus sequence types employing the pubMLST database (http://pubmlst.org/cdifficile). All the analyses were conducted using the BioNumerics version 7.6 platform (Applied Maths NV, Sint-Martens-Latem, Belgium).

### Descriptive Analyses

Organisms with the same ribotype and/or cgMLST that were isolated at different study time points from the same subject were assumed to represent a single strain. Likewise, organisms with the same ribotype and/or cgMLST isolated from different members of the same household were assumed to represent a single strain. When there was discordance between ribotype and cgMLST strain assignment, priority was given to the latter.

Strains were designated as “shared” if isolated from >1 family member, and “unique” if the organism was detected from only a single member within the family group. The pattern of colonization within each subject was assigned using the definitions employed previously in longitudinally studied hospitalized adults [6]. Specifically, colonization within a given subject that was detected on a single study time point was judged “transient”; colonization by strain-identical organisms detected on >1 study time point was designated “persistent,” including instances in which there were intervening samples that were negative. If colonization was detected only on the first or last time point, the duration of colonization was deemed “indeterminate.” When individual subjects were colonized by >1 strain, these patterns assignments were assessed for each strain separately.

## RESULTS

Thirty families were studied between September 2021 and January 2023. All infants and mothers participated, including 3 sets of infant twins; additionally, 19 fathers took part in the study. Nineteen families submitted all 8 samples, and 25 submitted 4 or more samples. All parents reported that they and their infants had no significant health issues. Among the infants, 19 (65.5%) had been born vaginally, and all but 2 were full term. Thirteen (43.3%) lived with 1 sibling, and 9 (29.9%) lived with 2 or more. Fifteen families (60% of respondents) owned a dog or a cat. At enrollment, 4 (13.8%) attended day care and 18 (62.0%) were being fed breast milk either exclusively or with other nutrition, although percentages of both these characteristics changed as the infants grew older.

*Clostridioides difficile*, including both toxigenic and nontoxigenic organisms, was isolated during the course of the study in at least 1 member in 28 of the 30 families. No parent or infant was diagnosed with CDI. In total, 225 organisms were cultivated; 191 could be recovered and strain-typed either by PCR ribotyping, cgMLST, or both. Strains were shared in 17 of the 28 families harboring *C difficile* (Table 1). In 3 of these families, 2 different strains were shared; hence, strains were shared in 20 instances. Shared strains were derived from 8 different ribotypes, with 2 (F106 and F014-020) predominating. (The distribution of all ribotypes isolated in this study is presented in Supplementary Table 2). Ninety percent of shared strains were toxigenic. The infant and at least 1 adult family member were implicated in 17 of these 20 strain sharing instances. In the remaining instances, the organism was shared between 2 adults without identification in the baby (once) or between twin infants without an identified adult (twice). The infant was usually the family member in whom the organism was first detected. Specifically, in the 17 instances in which an infant and at least 1 adult family member shared the same *C difficile* strain, the infant was detected first in 13, either alone or simultaneously with an adult (Table 1).

**Table 1.** Distribution of Toxigenic + Nontoxigenic Isolates

Strain designation	Families^a^	Instances
Shared strains^b^	17	20
Infant + adult	16	17
** ** Infant (± adult) detected first	…	13
** ** Adult only detected first	…	4
Adult + adult	1	1
Twin + twin	2	2
Unique strains^c^	25	49
Infant	18	27
Parent	18	22

^a^Fifteen families harbored both shared and unique strains.

^b^Strains were designated as “shared” if they were detected in >1 family member.

^c^Strains were designated as “unique” if they were detected in only a single family member.

Twenty-five families harbored 49 additional *C difficile* isolates that were unique, that is, they were identified in only 1 family member (Table 1). Twenty-seven of these unique isolates were cultivated from infants, and 22 were identified in adults. Unique strains were derived from 18 different ribotypes. There were 9 isolates belonging to ribotype FP313, but the remainder were distributed in small numbers among other ribotypes. Forty-three percent of unique isolates were toxigenic.

When only toxigenic strains were considered, 22 families were colonized. The frequency of strain sharing within these families was similar to that noted when all *C difficile* isolates, toxigenic and nontoxigenic, were included (Supplementary Table 3). Specifically, 15 of these 22 families harbored 18 shared strains, with infants identified in 15 of these instances. Similar to the longitudinal pattern noted when both toxigenic and nontoxigenic organism were considered, infants were identified first in most of these instances, with or without a concomitantly identified adult. The numbers of unique isolates were substantially reduced when only toxigenic strains were considered, reflective of the large proportion of unique strains that were nontoxigenic (Supplementary Table 3).

**Figure 1.**
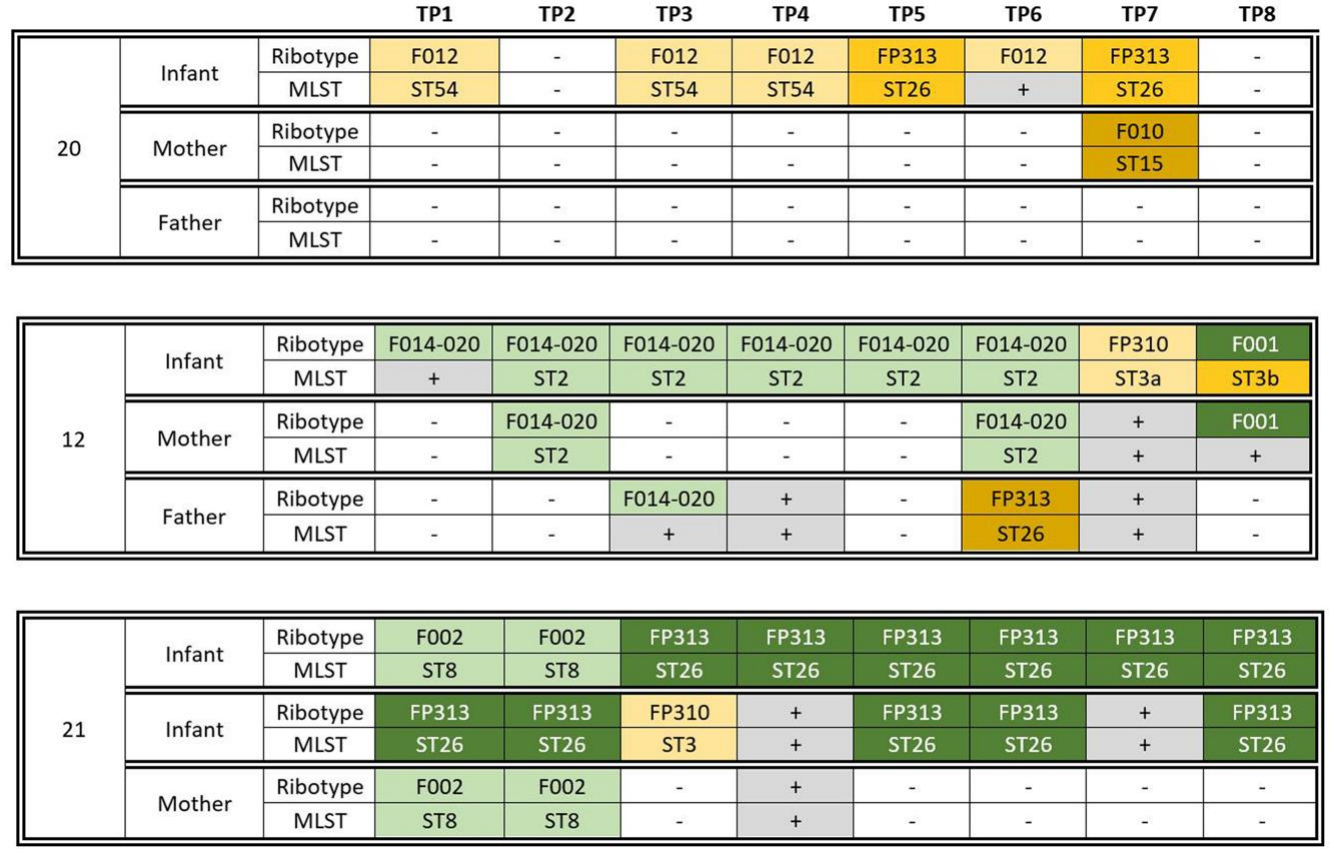
Pattern of *Clostridioides difficile* excretion in 3 typical families, labeled 20, 12, and 21 (corresponding to their designations in Supplementary Figure 1*A*). Each family includes 3 members: infant, mother, and father in families 20 and 12, and twin infants and mother in family 21. Each row represents biweekly culture results at each of the 8 study time points (TP), with the top half of each row reporting the polymerase chain reaction ribotype designation and the bottom half reporting the core genome multilocus sequence type (MLST). Boxes in gray indicate that the culture was positive but the isolate could not be recovered for strain testing. Shared isolates are colored green, with different shades indicating different strains. Unique isolates are colored orange, with different shades indicating different strains.

When observed longitudinally, the epidemiology of *C difficile* proved to be complex, shifting, and ephemeral. With both shared and unique patterns of colonization, organisms regularly appeared and disappeared over the period of observation (Figure 1; the pattern for each of the 28 families harboring *C difficile*, including all isolates and only toxigenic isolates, is presented in Supplementary Figure 1*A* and 1*B*). In a representative family excreting only unique organisms, for example (Figure 1, family 20), the infant was colonized by 2 different strains, while the mother was found positive by a third. In a typical family harboring shared strains (Figure 1, family 12), 1 strain (F014-020/ST2) was isolated from all 3 family members, and a second (F001/ST3b, although the MLST group could be determined for only 1 of the isolates) likely was shared by the infant and mother. Two additional strains (1 from the baby, 1 from the father) were unique. Strain sharing was even more striking in a family raising infant twins (Figure 1, family 21).

Of the 20 shared strains isolated from infants, including both toxigenic and nontoxigenic strains, 15 were persistently excreted, and of 16 shared strains cultivated from mothers, 8 were persistent (Table 2). Persistence of shared strains in fathers was uncommon. By contrast, the duration of colonization among the unique strains was substantially shorter than that observed in the shared isolates. Among infants, colonization in 14 of 27 instances in which a unique isolate was detected was transient, and in 6 colonization was indeterminate. Similarly, in the majority of instances in which a unique strain was identified in the parent, colonization was transient (Table 2).

**Table 2.** Pattern of Colonization of Shared and Unique Isolates

Strain designation	No.	Persistent	Transient	Indeterminate
Shared strains				
Infant	20	15	4	1
Mother	16	8	5	3
Father	10	2	6	2
Unique strains				
Infant	27	7	14	6
Mother	16	0	11	5
Father	6	1	4	1

Colonization was deemed persistent if *Clostridioides difficile* was isolated from the same subject on >1 study time point. Colonization was designated as transient if *C difficile* was isolated from the same subject on only a single study time point. Duration was indeterminate if the organism was isolated from only the first or last study time point.

To additionally assess the burden of *C difficile* in families caring for a healthy infant, cross-sectional prevalence of *C difficile* positivity, including both toxigenic and nontoxigenic organisms, was determined for each of the initial 4 study time points, corresponding to the infants’ age 5 to approximately 6.5 months, since these were the visits with the most consistent participation. The prevalence among the infants ranged from 50.0% to 71.4%, consistent with our previously published data indicating a high frequency of asymptomatic excretion in babies peaking around age 6 months [4] (Table 3). The prevalence in adult caregivers, while not as high, was still notable: The prevalence in mothers ranged from 20.0% to 40.0%, and the prevalence in the fathers ranged from 20.0% to 37.5% (Table 3), compared with the 2%–8% prevalence reported in cross-sectional studies of other nonhospitalized, unselected healthy adult populations [2, 9]. When the cross-sectional prevalence calculations were confined to toxigenic organisms, the proportions were lower, as expected, but they still mostly exceeded those recorded in previously assessed healthy populations. Infant prevalence ranged from 26.3% to 57.1%, prevalence among mothers ranged from 8.6% to 30.0%, and prevalence among fathers ranged from 6.2% to 25.0%, although the numbers for each study visit were small (Supplementary Table 4)

**Table 3.** Cross-Sectional Prevalence of *Clostridioides difficile* Colonization (Toxigenic + Nontoxigenic)

Study Time Point	1	2	3	4
Median infant age (range), mo	5.07 (4.30–7.10)	5.63 (4.67–7.63)	6.10 (5.10–8.13)	6.53 (5.73–8.60)
Infant	19/33 (57.6%)	15/30 (50.0%)	18/29 (62.1%)	20/28 (71.4%)
Mother	12/30 (40.0%)	10/26 (38.5%)	8/26 (30.8%)	5/25 (20.0%)
Father	5/19 (26.3%)	5/19 (26.3%)	6/16 (37.5%)	3/15 (20.0%)

Data are presented as no./No. (%).

## DISCUSSION

The current study demonstrated that sharing of *C difficile* isolates in families caring for an infant is a common event. Because each sampling time point was separated by approximately 2 weeks, our study was unable to demonstrate the directionality of transmission with certainty. Infants were commonly identified earliest within a given family, and the infrequency of exclusive adult colonization detected as the first event suggests, but does not prove, that the infant usually was the index. Indeed, the observed longitudinal pattern of colonization supports that in some families, transmission was multidirectional, and our data additionally allow that some or all members may have been colonized by a common source not identified by the protocol. However, regardless of how the organism initially entered the household, this study indicates that the prevalence of *C difficile* colonization was unexpectedly high in families caring for a healthy infant. The common occurrence of unique strain excretion among both infants and adults was unexplained, but transmission of these organisms among family members may have been undetected by the interrupted sampling schedule and the transient colonization pattern noted among most unique strains.

Other investigators have identified several putative sources of *C difficile* in the community, including outpatient clinics, particularly high-risk settings caring for complicated patients [10–12] and home-dwelling adults who previously had been diagnosed with CDI [13–15]. More recent analyses of large population datasets indicate that previously hospitalized adults who had no diagnosis of CDI while in the hospital also increase the risk of CA-CDI in their household contacts [16]. The latter finding highlights that persons with undetected *C difficile* excretion may pose a particular risk to their community contacts.

To this list of potential sources of CA-CDI, the data from the current study support adding similarly undetected infant *C difficile* excreters and their families. The role of asymptomatically colonized infants in the CA-CDI epidemic has been speculated, but the evidence implicating them as a reservoir has been indirect. We [4] and others [17–19] have shown that strains isolated from infants overlap with those detected in adults with CDI within the same geographic area. Additionally, Chitnis and colleagues [10], surveying CA-CDI between 2009 and 2011 through the Centers for Disease Control and Prevention–sponsored Emerging Infections Program, found an association of infant contact and acquisition of CA-CDI among adults who had had no high-risk outpatient exposure. In each of these cases, however, transmission from infant to adult was not measured directly. The current study, therefore, strengthens the speculated role of infants in CA-CDI epidemiology by providing concrete evidence bridging *C difficile*–colonizing infant and adult populations. Further evidence will need to be established (for example, CA-CDI in secondary or tertiary contacts of the parents) before infants’ families can be established as a community reservoir with confidence.

The clinical and epidemiological impact of infants in perpetuating CA-CDI in the community is likely mitigated by the health of most of their caregivers, who are largely comprised of medically uncomplicated young adults in whom CA-CDI is an uncommon event. Hence, even if transmission from infant to parent and then from the parent to a subsequent adult contact is frequent, the development of *C difficile* colitis may be uncommon. Indeed, no adult participant in the current study was diagnosed with CA-CDI. On the other hand, infant care is nearly universal at some point in most households, and transmission of the organism to a household contact and beyond may occur sufficiently frequently to render the baby and his or her family an important reservoir for CDI in the community, even if the risk of acquiring CA-CDI among their contacts at the individual level is small.

Some limitations of this study should be noted. First, the generalizability of our findings awaits study of other populations. Our survey was conducted at a single suburban pediatric office and was comprised of a small convenience sample, and willingness to enroll may have injected undetected selection bias into the study sample. The frequency and dynamics of familial *C difficile* transmission may be different in other settings, such as close-quartered or economically disadvantaged communities. Second, while broth enrichment is very sensitive in detecting *C difficile*, we cannot exclude the possibility that some subjects may have harbored the organism below the level of detection. Third, strain typing by ribotyping and cgMLST was sometimes discordant. However, these strain-typing techniques are inherently different, and while there frequently is agreement between PCR ribotyping and cgMLST assignment, it is not universal [20]. Hence, some discordance was to be expected. Moreover, both strain-typing techniques were applied to separate colonies cultivated from a given subject, and co-excretion of different strains, as has been documented by others [17], was possible. Fourth, we chose to study intrafamilial *C difficile* strain sharing at an infant age when colonization was particularly prevalent, to increase the likelihood that we would detect strain sharing if it occurred. The pattern of strain sharing may have been different if we had chosen other periods in the infants’ first year of life. Fifth, it is possible that some of the family samples were cross-contaminated by the adult shipping the samples. Out of concern for this possibility, we asked the last 8 families to submit a control wipe, which was subjected to broth enrichment on arrival to laboratory. Of 41 control samples, 1 was positive, suggesting that some preculture contamination was possible, though infrequent. Sixth, factors other than infant contact may have contributed to the unexpectedly high frequency of *C difficile* excretion in the parents. Information regarding antibiotic and antacid use in the adults was collected during this study, for example, but the numbers were too small to render meaningful conclusions.

The findings of the current study represent an important step in defining the epidemiological role of infants in CA-CDI in older persons. The observations derived by our study provide an impetus to define the source of the colonizing infant organisms, as intervention at the point of baby acquisition may provide additional barriers to the spread of the organism outside the hospital. It is likely that community reservoirs of *C difficile* are multiple; controlling intrafamilial sharing of the organism in households caring for an infant may prove to be one of several interventions necessary to control this threat to the public health.

## Supplementary Data

Supplementary materials are available at *Open Forum Infectious Diseases* online. Consisting of data provided by the authors to benefit the reader, the posted materials are not copyedited and are the sole responsibility of the authors, so questions or comments should be addressed to the corresponding author.

## Supplementary Material

ofae299_Supplementary_Data

## Notes

***Acknowledgments.*** The authors acknowledge the support and help of Rajashri Rasal, the lead study coordinator of the Rainbow Critical Care Clinical Research Group.

***Author contributions.*** C. M. was the principal coordinator of the study; was in charge of enrollment, obtaining consent, monitoring and encouraging adherence among the subjects, monitoring receipt of stool samples, and resolving any internal inconsistencies in the study dataset; and wrote the first drafts of the manuscript. J. A. C. conceived the study aims and design. N. M. analyzed the study datasets. G. G. developed the enrollment plan. L. M. introduced the family to the study, encouraged enrollment after distributing a study information sheet, and provided the contact information of potentially interested families. A. L. J. and J. M. H. contributed to culturing the stool samples in the Donskey research microbiology laboratory. H. C. planned and performed the whole genome sequencing (WGS) of the isolates. P. C. and M. H. assisted in the planning and performing of WGS of the isolates. C. J. oversaw WGS analysis and MLST assignment. J. L. C. oversaw all culturing and species identification procedures and further developed and devised the laboratory datasets. C. J. D. contributed to study design, procedures, and data analysis and interpretation throughout the study. P. T. conceived the study aims and design, oversaw all elements of the study, analyzed the data, and contributed to the writing of the manuscript. All authors reviewed and approved the final manuscript.

***Financial support*.** This study was supported by grants from the Rainbow Board of Trustees, and by the US Department of Veterans Affairs Office of Research and Development as part of funding for VASeqCURE, which in turn received funding from American Rescue Plan Act funds. All genome sequences generated by this project have been submitted to NCBI. BioProject ID: PRJNA1111124.
