# Supplementary material for: Molecular Epidemiology of Clostridioides difficile Colonization in Families With Infants
Source: Open Forum Infect Dis. 2024 Jun 10;11(6):ofae299. doi: 10.1093/ofid/ofae299 (PMC11192056; doi:10.1093/ofid/ofae299)
Supplement: ofae299_Supplementary_Data [file ofae299_supplementary_data.zip › Marlow OFID Supplement Corrected Clean 6-11-24.docx]

**Supplemental Materials**

**Table S1.** Detailed Description of WGS Methodology

**Table S2.** Distribution of Ribotypes Isolated from the Total Study Cohort

**Table S3.** Distribution of Isolates, Considering Only Toxigenic Organisms

**Figure S1.** Pattern of *C. difficile* Excretion for the Entire Study Cohort. Figure S1A, All Isolates; Figure S1B, Only Toxigenic Isolates

**Table S4.** Prevalence of *C. difficile* Isolation for the First 4 Study Time Points, Considering Only Toxigenic Isolates

**Table S1. Detailed Description of WGS Methodology**

For WGS-based core genome multilocus sequence typing (cgMLST) ^8^, *C. difficile* isolates were cultivated on anaerobic blood agar plates overnight. Bacterial DNA extraction was accomplished using QIAamp DNA Micro Kit (Qiagen, Hilden, Germany) and DNA library was prepared using the Nextera DNA Prep Kit (Illumina, San Diego, CA). Paired end reads (2x150bp) were generated using the Illumina NextSeq mid output reagent kit and run on NextSeq 550 instrument (Illumina, San Diego, CA). All the analyses were conducted using the BioNumerics version 7.6 platform (Applied Maths NV, Sint-Martens-Latem, Belgium). De novo assembly was completed using SPAdes genome assembler on a cloud-based platform (Applied Maths, Austin, TX). To ensure highest quality of analysis, we aimed for 100x coverage with majority of the isolates (110 out of 160) being over 100x threshold. Forty-six out of the remaining 50 isolates were over 60x coverage. The remaining 4 isolates were between 40x to 60x coverage. cgMLST was performed using BioNumerics calculation engine and the isolates were assigned pubMLST sequence type for comparison (database http://pubmlst.org/cdifficile). By default, BioNumerics calculation engine analyzed 1999 core loci for *C. difficile*. The metrics from the sequence run such as number of contigs, genome length, N50, and genome fold coverage is provided in the table below (S1) for each isolate. Additionally, all the sequences have been deposited into publicly available database (NCBI).

| Family group | Subject Type | Subject ID | Sample | PubMLST ST | N50 | Number of Contigs | Genome fold-coverage | Genome Length |
| --- | --- | --- | --- | --- | --- | --- | --- | --- |
| 1 | Baby | 107 | 1 | ST42 | 460375 | 43 | 130 | 4018747 |
| 1 | Baby | 107 | 3 | ST42 | 485185 | 45 | 85 | 4019601 |
| 1 | Mother | 108 | 3 | ST42 | 379768 | 43 | 111 | 4018978 |
| 1 | Father | 109 | 1 | ST42 | 423257 | 42 | 122 | 4019006 |
| 2 | Mother | 105 | 1 | ST42 | 162245 | 78 | 66 | 4011187 |
| 2 | Father | 106 | 3 | ST37 | 277368 | 44 | 85 | 4096016 |
| 3 | Baby | 116 | 1 | ST42 | 455844 | 42 | 142 | 4020292 |
| 3 | Baby | 116 | 3 | ST42 | 455844 | 42 | 132 | 4020006 |
| 3 | Infant | 116 | 5 | ST42 | 460236 | 42 | 129 | 4019394 |
| 3 | Baby | 116 | 7 | ST42 | 435112 | 43 | 126 | 4020298 |
| 3 | Infant | 116 | 8 | ST42 | 306537 | 66 | 82 | 4010879 |
| 3 | Mother | 117 | 1 | ST42 | 379630 | 43 | 117 | 4019077 |
| 3 | Mother | 117 | 8 | ST2 | 180180 | 112 | 101 | 4135745 |
| 4 | Infant | 119 | 1 | ST3 | 137952 | 174 | 84 | 4149099 |
| 4 | Infant | 119 | 2 | ST3 | 257768 | 95 | 124 | 4183345 |
| 4 | Infant | 119 | 3 | ST3 | 140699 | 151 | 197 | 4181865 |
| 4 | Baby | 119 | 4 | ST3 | 211620 | 110 | 114 | 4182268 |
| 4 | Mother | 120 | 4 | ST3 | 127808 | 162 | 88 | 4141715 |
| 5 | Baby | 122 | 1 | ST15 | 395890 | 63 | 116 | 4090503 |
| 5 | Baby | 122 | 3 | ST15 | 547307 | 58 | 142 | 4091602 |
| 5 | Baby | 122 | 5 | ST15 | 395980 | 67 | 119 | 4091658 |
| 5 | Baby | 122 | 6 | ST15 | 547307 | 60 | 124 | 4090665 |
| 5 | Infant | 122 | 7 | ST15 | 178695 | 103 | 63 | 4078929 |
| 5 | Infant | 122 | 8 | ST15 | 235943 | 100 | 87 | 4078915 |
| 5 | Mother | 123 | 1 | ST2 | 160977 | 110 | 76 | 4139655 |
| 5 | Mother | 123 | 3 | ST15 | 547307 | 64 | 131 | 4092373 |
| 5 | Mother | 123 | 6 | ST15 | 235943 | 97 | 78 | 4080105 |
| 5 | Father | 124 | 1 | ST2 | 160977 | 102 | 105 | 4143539 |
| 5 | Father | 124 | 2 | N/A | 150303 | 99 | 97 | 4471810 |
| 5 | Father | 124 | 3 | ST15 | 235943 | 99 | 72 | 4080408 |
| 5 | Father | 124 | 8 | ST26 | 193755 | 113 | 70 | 4190987 |
| 6 | Infant | 125 | 2 | ST2 | 190060 | 70 | 113 | 4158697 |
| 6 | Infant | 125 | 3 | ST2 | 160977 | 108 | 70 | 4138658 |
| 6 | Mother | 126 | 1 | ST2 | 189960 | 70 | 100 | 4157103 |
| 6 | Mother | 126 | 3 | ST3 | 137952 | 178 | 198 | 4150705 |
| 8 | Baby | 131 | 1 | ST2 | 235108 | 42 | 113 | 4076691 |
| 8 | Infant | 131 | 2 | ST3 | 137952 | 179 | 55 | 4146481 |
| 8 | Infant | 131 | 3 | ST2 | 187946 | 91 | 80 | 4060824 |
| 8 | Infant | 131 | 4 | ST2 | 203592 | 41 | 120 | 4077404 |
| 8 | Infant | 131 | 5 | ST2 | 138039 | 109 | 200 | 4111724 |
| 8 | Infant | 131 | 6 | ST2 | 198043 | 60 | 92 | 4123179 |
| 8 | Infant | 131 | 7 | ST2 | 160977 | 116 | 200 | 4112834 |
| 8 | Mother | 132 | 1 | ST3 | 137952 | 179 | 65 | 4150438 |
| 8 | Mother | 132 | 2 | ST3 | 222795 | 106 | 114 | 4182717 |
| 8 | Father | 133 | 1 | ST2 | 187945 | 87 | 203 | 4065889 |
| 9 | Baby | 134 | 2 | ST11 | 145679 | 62 | 115 | 3895812 |
| 9 | Infant | 134 | 4 | ST26 | 264757 | 56 | 109 | 4214464 |
| 9 | Infant | 134 | 6 | ST26 | 264425 | 59 | 104 | 4213230 |
| 9 | Infant | 134 | 7 | ST26 | 156777 | 118 | 196 | 4194179 |
| 9 | Infant | 134 | 8 | ST110 | 203601 | 53 | 89 | 4103115 |
| 9 | Mother | 135 | 1 | ST14 | 197994 | 76 | 115 | 4294536 |
| 9 | Mother | 135 | 2 | ST26 | 255872 | 59 | 120 | 4215132 |
| 9 | Mother | 135 | 8 | ST63 | 215284 | 67 | 58 | 4038467 |
| 10 | Baby | 136 | 2 | ST2 | 203593 | 66 | 122 | 4149218 |
| 10 | Infant | 136 | 3 | ST2 | 180180 | 105 | 116 | 4134757 |
| 10 | Infant | 136 | 4 | ST2 | 198043 | 70 | 111 | 4147597 |
| 10 | Infant | 136 | 5 | ST2 | 180115 | 105 | 129 | 4135310 |
| 10 | Infant | 136 | 6 | ST2 | 203593 | 65 | 112 | 4148501 |
| 10 | Infant | 136 | 7 | ST3 | 161849 | 144 | 197 | 4182484 |
| 10 | Infant | 136 | 8 | ST3 | 249818 | 47 | 139 | 4123124 |
| 10 | Mother | 137 | 2 | ST2 | 215548 | 43 | 128 | 4076936 |
| 10 | Mother | 137 | 6 | ST2 | 180115 | 111 | 119 | 4136195 |
| 10 | Father | 138 | 6 | ST26 | 156777 | 122 | 115 | 4196247 |
| 12 | Baby | 147 | 5 | ST28 | 423263 | 42 | 127 | 4117605 |
| 12 | Baby | 147 | 6 | ST42 | 455844 | 44 | 109 | 4019339 |
| 12 | Father | 149 | 5 | ST28 | 423257 | 48 | 121 | 4115500 |
| 12 | Father | 149 | 6 | ST28 | 423263 | 45 | 116 | 4115515 |
| 13 | Baby | 152 | 3 | ST28 | 485211 | 42 | 132 | 4115303 |
| 13 | Infant | 152 | 4 | ST28 | 208085 | 66 | 172 | 4119791 |
| 13 | Infant | 152 | 8 | ST28 | 208041 | 69 | 74 | 4104416 |
| 13 | Mother | 153 | 3 | ST28 | 208041 | 72 | 72 | 4105793 |
| 15 | Infant | 159 | 1 | ST54 | 141377 | 118 | 96 | 4242080 |
| 15 | Infant | 159 | 3 | ST54 | 211881 | 72 | 117 | 4269429 |
| 15 | Baby | 159 | 4 | ST54 | 187490 | 119 | 98 | 4238728 |
| 15 | Infant | 159 | 5 | ST26 | 193754 | 114 | 72 | 4286462 |
| 15 | Infant | 159 | 7 | ST26 | 193754 | 112 | 65 | 4198296 |
| 15 | Mother | 160 | 7 | ST15 | 230123 | 61 | 78 | 3986847 |
| 16 | Infant | 150 | 7 | ST8 | 232687 | 78 | 40 | 4135831 |
| 16 | Mother | 151 | 2 | ST42 | 455844 | 41 | 114 | 4019396 |
| 18 | Infant | 128 | 1 | ST2 | 160977 | 99 | 109 | 4143489 |
| 18 | Infant | 128 | 2 | ST110 | 189891 | 108 | 124 | 4139339 |
| 18 | Infant | 128 | 4 | ST110 | 198033 | 67 | 128 | 4149233 |
| 18 | Infant | 128 | 6 | ST110 | 198033 | 66 | 128 | 4151651 |
| 18 | Infant | 128 | 7 | ST110 | 160954 | 113 | 126 | 4135879 |
| 18 | Mother | 129 | 7 | ST110 | 137994 | 106 | 62 | 4136926 |
| 18 | Father | 130 | 1 | ST8 | 227985 | 70 | 86 | 4123584 |
| 18 | Father | 130 | 2 | ST8 | 149972 | 75 | 114 | 4113824 |
| 18 | Father | 130 | 3 | ST8 | 214557 | 86 | 200 | 4116131 |
| 18 | Father | 130 | 4 | ST8 | 135594 | 82 | 68 | 4112224 |
| 18 | Father | 130 | 5 | ST8 | 217779 | 70 | 72 | 4114174 |
| 18 | Father | 130 | 6 | ST8 | 217779 | 75 | 76 | 4113970 |
| 18 | Father | 130 | 7 | ST8 | 214614 | 87 | 200 | 4115441 |
| 18 | Father | 130 | 8 | ST8 | 153365 | 77 | 78 | 4110923 |
| 20 | Infant | 144 | 1 | ST41 | 128648 | 86 | 202 | 4076720 |
| 20 | Infant | 144 | 3 | ST41 | 137437 | 78 | 61 | 4074374 |
| 20 | Infant | 144 | 4 | ST41 | 200636 | 64 | 118 | 4083745 |
| 20 | Infant | 144 | 5 | ST41 | 137433 | 78 | 97 | 4075335 |
| 20 | Infant | 144 | 6 | ST41 | 273170 | 57 | 118 | 4084097 |
| 20 | Infant | 144 | 7 | ST41 | 168308 | 72 | 58 | 4073119 |
| 20 | Infant | 144 | 8 | ST41 | 137433 | 80 | 116 | 4074953 |
| 20 | Father | 146 | 5 | ST41 | 137433 | 76 | 110 | 4075607 |
| 20 | Father | 146 | 6 | ST41 | 137433 | 79 | 121 | 4076344 |
| 21 | Infant | 162 | 1 | ST8 | 150276 | 72 | 144 | 4030894 |
| 21 | Infant | 162 | 2 | ST8 | 366082 | 47 | 129 | 4039887 |
| 21 | Infant | 162 | 3 | ST26 | 193755 | 113 | 74 | 4189952 |
| 21 | Infant | 162 | 4 | ST26 | 264757 | 60 | 90 | 4211654 |
| 21 | Infant | 162 | 5 | ST26 | 193755 | 114 | 71 | 4190885 |
| 21 | Infant | 162 | 6 | ST26 | 193755 | 120 | 65 | 4187950 |
| 21 | Infant | 162 | 7 | ST26 | 247733 | 76 | 127 | 4213878 |
| 21 | Infant | 162 | 8 | ST26 | 193755 | 114 | 76 | 4189407 |
| 21 | Infant | 163 | 1 | ST26 | 193755 | 112 | 135 | 4277786 |
| 21 | Infant | 163 | 2 | ST26 | 193755 | 107 | 83 | 4186697 |
| 21 | Infant | 163 | 3 | ST3 | 167122 | 125 | 143 | 4171036 |
| 21 | Infant | 163 | 5 | ST26 | 193755 | 113 | 61 | 4275047 |
| 21 | Infant | 163 | 6 | ST26 | 193755 | 115 | 62 | 4186355 |
| 21 | Infant | 163 | 8 | ST26 | 177342 | 123 | 193 | 4278008 |
| 21 | Mother | 164 | 1 | ST8 | 182169 | 70 | 101 | 4029315 |
| 21 | Mother | 164 | 2 | ST 8 | 182169 | 71 | 109 | 4031044 |
| 22 | Infant | 168 | 4 | ST 28 | 183273 | 88 | 113 | 4045204 |
| 22 | Infant | 168 | 5 | ST28 | 158658 | 75 | 78 | 4045220 |
| 22 | Infant | 168 | 6 | ST28 | 158658 | 79 | 77 | 4052318 |
| 22 | Infant | 168 | 7 | ST28 | 158658 | 98 | 203 | 4054174 |
| 22 | Infant | 168 | 8 | ST28 | 158658 | 102 | 203 | 4055829 |
| 22 | Mother | 169 | 7 | ST28 | 166356 | 68 | 154 | 4063570 |
| 22 | Father | 170 | 3 | ST26 | 195043 | 87 | 98 | 4209881 |
| 22 | Father | 170 | 7 | ST28 | 231098 | 68 | 82 | 4063404 |
| 24 | Infant | 177 | 2 | ST42 | 162296 | 87 | 198 | 4173857 |
| 24 | Infant | 177 | 4 | ST592 | 219924 | 63 | 132 | 4037059 |
| 24 | Infant | 177 | 5 | ST592 | 220184 | 58 | 171 | 4084282 |
| 24 | Infant | 177 | 6 | ST592 | 220212 | 60 | 153 | 4088326 |
| 24 | Infant | 177 | 7 | ST15 | 233143 | 53 | 205 | 4028690 |
| 24 | Infant | 177 | 8 | ST15 | 192966 | 91 | 202 | 4063933 |
| 24 | Father | 179 | 5 | ST42 | 162252 | 99 | 197 | 4163600 |
| 25 | Infant | 180 | 1 | ST2 | 160977 | 91 | 202 | 4070576 |
| 25 | Infant | 180 | 2 | ST42 | 162252 | 100 | 198 | 4164455 |
| 25 | Infant | 180 | 3 | ST2 | 160977 | 88 | 202 | 4070190 |
| 25 | Infant | 180 | 4 | ST2 | 137753 | 98 | 202 | 4068935 |
| 25 | Infant | 180 | 5 | ST2 | 162699 | 93 | 202 | 4068909 |
| 25 | Infant | 180 | 6 | ST2 | 173361 | 72 | 184 | 4083184 |
| 25 | Infant | 180 | 7 | ST2 | 173354 | 86 | 202 | 4070542 |
| 25 | Infant | 180 | 8 | ST2 | 160977 | 94 | 202 | 4068681 |
| 25 | Infant | 181 | 1 | ST2 | 187988 | 77 | 127 | 4071850 |
| 25 | Infant | 181 | 2 | ST2 | 160977 | 89 | 202 | 4069381 |
| 25 | Infant | 181 | 3 | ST2 | 137754 | 101 | 202 | 4066556 |
| 25 | Infant | 181 | 4 | ST2 | 173354 | 91 | 202 | 4065812 |
| 25 | Infant | 181 | 5 | ST2 | 160977 | 88 | 202 | 4065924 |
| 25 | Infant | 181 | 6 | ST2 | 137754 | 92 | 134 | 4064883 |
| 25 | Infant | 181 | 7 | ST2 | 198043 | 70 | 200 | 4079988 |
| 25 | Infant | 181 | 8 | ST2 | 187946 | 88 | 120 | 4067468 |
| 25 | Mother | 182 | 2 | ST28 | 158658 | 101 | 203 | 4054723 |
| 26 | Infant | 183 | 1 | ST42 | 306537 | 80 | 205 | 4012330 |
| 26 | Mother | 184 | 1 | ST26 | 208891 | 91 | 189 | 4212624 |
| 27 | Infant | 185 | 1 | ST15 | 292114 | 47 | 207 | 3995993 |
| 27 | Infant | 185 | 2 | ST15 | 233134 | 39 | 87 | 3992780 |
| 28 | Infant | 187 | 1 | ST15 | 206420 | 98 | 203 | 4061010 |
| 28 | Infant | 187 | 2 | ST15 | 228403 | 80 | 149 | 4072356 |
| 28 | Infant | 187 | 4 | ST3 | 153630 | 144 | 104 | 4147505 |
| 28 | Infant | 187 | 5 | ST42 | 306638 | 62 | 182 | 4059301 |
| 28 | Infant | 187 | 8 | ST3 | 161849 | 124 | 148 | 4161475 |
| 28 | Mother | 188 | 2 | ST28 | 184045 | 71 | 153 | 4064687 |

**Table S2. Distribution of Ribotypes Isolated from the Entire Study Cohort**

| Ribotype | Number of Families |
| --- | --- |
|  |  |
| F001 | 1 |
| F002 | 4 |
| F010 | 6 |
| F012 | 2 |
| F014-20 | 9 |
| F017 | 1 |
| F078-126 | 1 |
| F106 | 14 |
| F116 | 1 |
| F137 | 1 |
| F255 | 1 |
| F310 | 8 |
| F313 | 10 |
| F370 | 1 |
| F407 | 1 |
| F427 | 1 |
| F452 | 2 |
| F494 | 1 |
| F502 | 1 |

| **Table S3. Distribution of Toxigenic Isolates** | | | |
| --- | --- | --- | --- |
| Strain Designation |  | Families^1^ | Instances |
| Shared Strains^2^ |  | 15 | 18 |
| Infant + Adult |  | 14 | 15 |
|  | Infant (+/- adult) detected first |  | 12 |
|  | Adult only detected first |  | 3 |
| Adult + Adult |  | 1 | 1 |
| Twin + Twin |  | 2 | 2 |
|  |  |  |  |
| Unique Strains^3^ |  | 14 | 26 |
| Infant |  | 11 | 15 |
| Parent |  | 9 | 11 |

1. 22 of the 30 enrolled families harbored toxigenic *C. difficile*
2. Strains were designated as “shared” if they were detected in >1 family member.
3. Strain were designated at “unique” if they were detected in only a single family member.

**Figure legend. Figure S1A, Longitudinal representation of the 28 families colonized by *C. difficile*, including both toxigenic and non-toxigenic organisms.**  Columns represent study time points 1 through 8. Isolates are represented by their PCR-ribotype designations and cgMLST assignments. Green coloring indicates isolates that were shared within families, with shades designating different strains. Yellow/brown coloring indicates unique isolates, in which intra-family sharing was not detected, with shades designating different strains. Gary coloring represents isolates that could not be strain-typed. Isolates labeled with a superscripted “B” were identified only by broth enrichment. **Figure S1B, Longitudinal representation of the same 28 families in Figure S1A, with non-toxigenic strains excluded.** Color designations are the same as Figure S4A; non-toxigenic isolates are denoted by the label “TOX NEG.” If any organism was found to be toxigenic by EIA testing of the isolate, it was assumed that all organisms from the same strain isolated from the same family were toxigenic, even if EIA results of some samples were negative.

**Table S4. Prevalence of *C. difficile* Isolation for the First 4 Study Time Points, Confined to only Toxigenic Isolates**

| Study Time Points | 1 | 2 | 3 | 4 |
| --- | --- | --- | --- | --- |
| Infant | 14/31 (45.2%) | 12/29 (41.4%) | 15/27 (55.6%) | 16/28 (57.1%) |
| Mother | 6/28 (21.4%) | 8/27 (30.0%) | 4/26 (15.4%) | 3/35 (8.6%) |
| Father | 3/18 (16.7%) | 2/15 (13.3%) | 4/16 (25.0%) | 1/15 (6.7%) |
