# Supplementary figures and images for: Molecular Epidemiology of Clostridioides difficile Colonization in Families With Infants
Source: Open Forum Infect Dis. 2024 Jun 10;11(6):ofae299. doi: 10.1093/ofid/ofae299 (PMC11192056; doi:10.1093/ofid/ofae299)

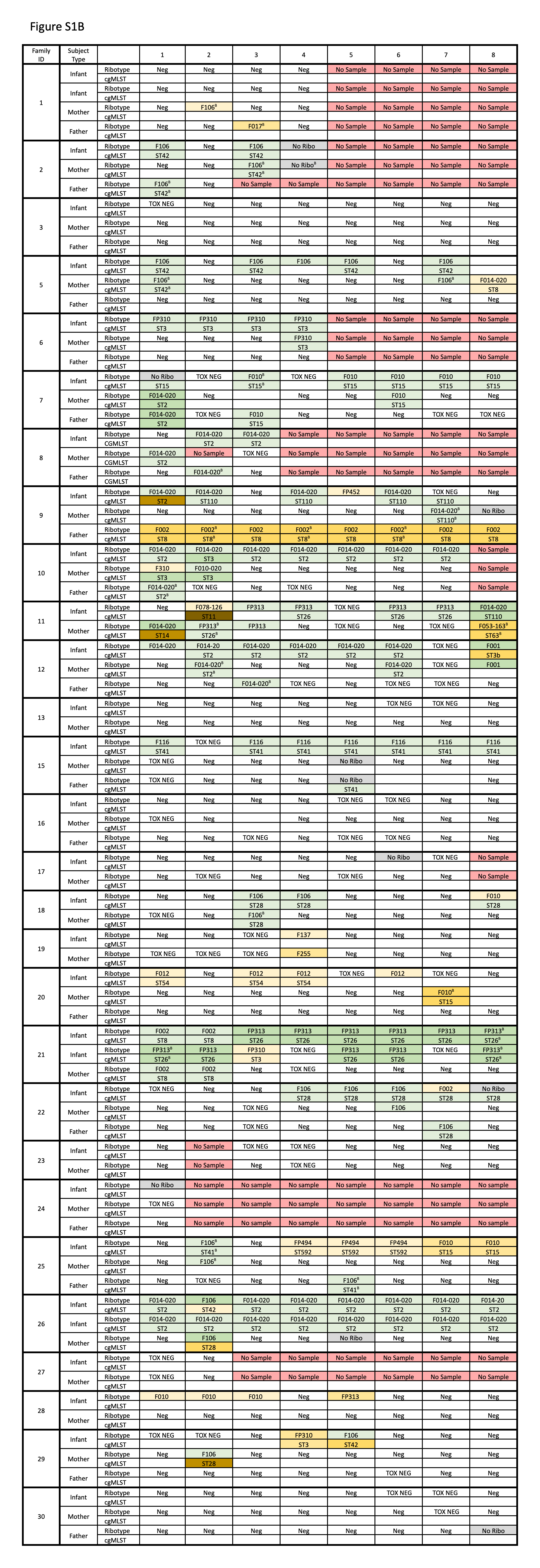

Supplement: ofae299_Supplementary_Data [file ofae299_supplementary_data.zip › Marlow OFID Figure S1B.jpg]

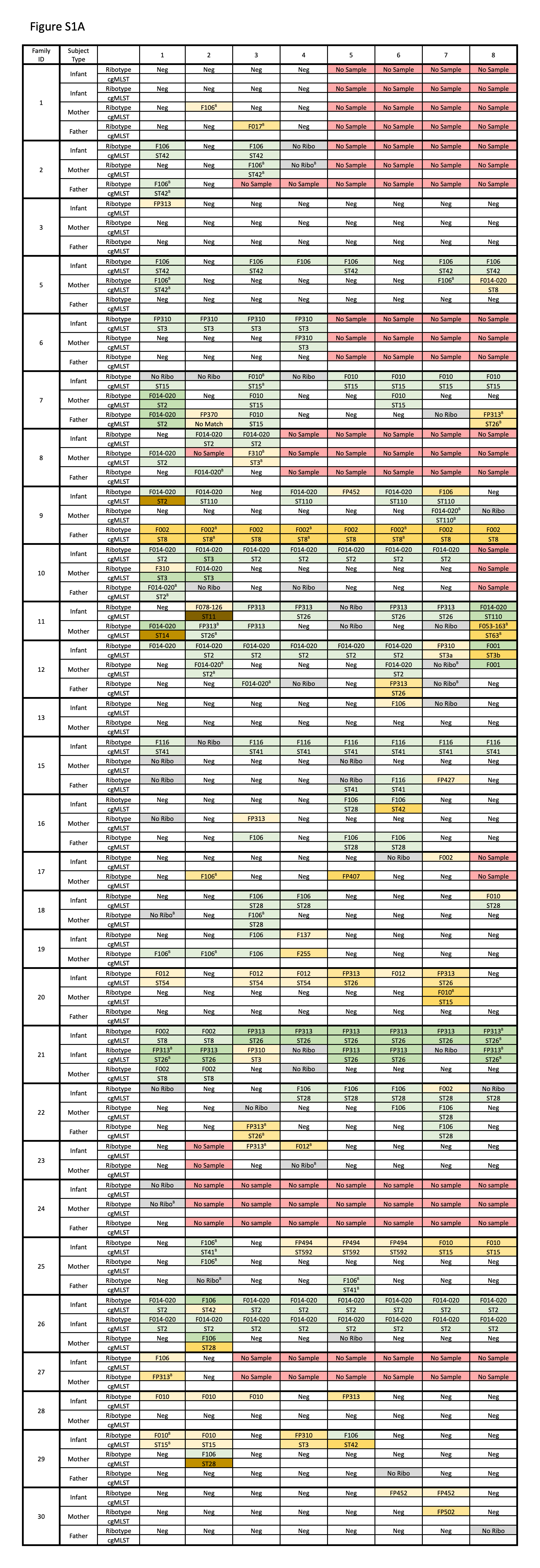

Supplement: ofae299_Supplementary_Data [file ofae299_supplementary_data.zip › Marlow OFID Figure S1A.jpg]
